# Regeneration of Critical Calvarial Bone Defects Using Bovine Xenograft, Magnesium-Enriched Bovine Xenograft and Autologous Dentin in Rats: Micro-CT, Gene Expression and Immunohistochemical Analysis

**DOI:** 10.3390/jfb15090270

**Published:** 2024-09-18

**Authors:** Marija Čandrlić, Ana Terezija Jerbić Radetić, Hrvoje Omrčen, Barbara Franović, Lara Batičić, Tamara Gulić, Tea Čaljkušić-Mance, Sanja Zoričić Cvek, Lucija Malešić, Željka Perić Kačarević, Olga Cvijanović Peloza

**Affiliations:** 1 Department of Integrative Dental Medicine, Faculty of Dental Medicine and Health Osijek, J.J. Strossmayer University of Osijek, 31000 Osijek, Croatia; marija.candrlic@fdmz.hr; 2 Department of Anatomy, Faculty of Medicine, University of Rijeka, Braće Branchetta 20/1, 51000 Rijeka, Croatia; ana.jerbic.radetic@uniri.hr (A.T.J.R.); barbara.franovic@medri.uniri.hr (B.F.); sanja.zoricic@medri.uniri.hr (S.Z.C.); lucija.malesic@gmail.com (L.M.); 3 Department of Clinical Microbiology, Teaching Institute of Public Health of Primorsko-Goranska County, Krešimirova 52a, 51000 Rijeka, Croatia; hrvoje.omrcen@zzjzpgz.hr; 4 Doctoral School of Biomedicine and Health, Faculty of Medicine, University of Rijeka, Braće Branchetta 20/1, 51000 Rijeka, Croatia; 5 Department of Medical Chemistry, Biochemistry and Clinical Chemistry, Faculty of Medicine, University of Rijeka, Braće Branchetta 20/1, 51000 Rijeka, Croatia; lara.baticic@uniri.hr; 6 Department of Physiology, Immunology and Pathophysiology, Faculty of Medicine, University of Rijeka, Braće Branchetta 20/1, 51000 Rijeka, Croatia; tamara.gulic@uniri.hr; 7 Department of Ophthalmology, Faculty of Medicine, University of Rijeka, Braće Branchetta 20/1, 51000 Rijeka, Croatia; tea.mance.caljkusic@uniri.hr; 8 Clinic for Ophthalmology, Clinical Hospital Center Rijeka, Krešimirova 42, 51000 Rijeka, Croatia; 9 Dental Clinic Rident, Franje Čandeka 39, 51000 Rijeka, Croatia; 10 Department of Anatomy, Histology, Embriology, Pathology Anatomy and Pathology Histology, Faculty of Dental Medicine and Health Osijek, J.J. Strossmayer University of Osijek, 31000 Osijek, Croatia; 11 Botiss Biomaterials GmbH, 15806 Zossen, Germany

**Keywords:** bovine xenograft, autologous dentin, critical calvaria size defect, magnesium, rats

## Abstract

The aim of this study was to evaluate the efficacy of autologous dentin (AD), bovine xenograft (BX) and magnesium-enriched bovine xenograft (BX + Mg) in the healing of critical cranial bone defects (CCBDs) in rats. Eighty male Wistar rats were divided into four groups: BX, BX + Mg, AD and the control group (no intervention). Eight mm CCBDs were created and treated with the respective biomaterials. Healing was assessed 7, 15, 21 and 30 days after surgery by micro-computed tomography (micro-CT), real-time polymerase chain reaction (RT-PCR) and immunohistochemical analysis. Micro-CT analysis showed that AD had the highest bone volume and the least amount of residual biomaterial at day 30, indicating robust bone formation and efficient resorption. BX + Mg showed significant bone volume but had more residual biomaterial compared to AD. RT-PCR showed that the expression of osteocalcin (OC), the receptor activator of nuclear factor κB (RANK) and sclerostin (SOST), was highest in the AD group at day 21 and vascular endothelial growth factor (VEGF) at day 15, indicating increased osteogenesis and angiogenesis in the AD group. Immunohistochemical staining confirmed intense BMP-2/4 and SMAD-1/5/8 expression in the AD group, indicating osteoinductive properties. The favorable gene expression profile and biocompatibility of AD and BX + Mg make them promising candidates for clinical applications in bone tissue engineering. Further research is required to fully exploit their potential in regenerative surgery.

## 1. Introduction

The formation of bone tissue, known as osteogenesis or morphogenesis, involves the development of bone tissue through a process that involves the differentiation of osteogenic cells into osteoblasts (a concept known as osteoinduction). For bone formation to occur, the osteoblasts and newly formed bone tissue require a suitable scaffold (a concept known as osteoconduction) that provides a porous structure that allows for the inward three-dimensional growth of bone tissue from the surface. Finally, osteogenesis essentially comprises two processes: the formation (osteoformation) and the breaking down (osteoresorption) of bone tissue. Both processes occur simultaneously and synergistically as they act in a balanced manner to create and maintain the tissue homeostasis of bone tissue according to functional requirements [1,2].

The formation of bone tissue involves a complex interplay of gene regulatory networks that orchestrate the differentiation of osteogenic cells into osteoblasts. These networks, particularly the SMAD proteins, play an important role in signaling through morphogenetic proteins (BMPs), which are essential for the regulation of osteogenesis and bone remodeling processes. BMPs promote the expression of osteogenic genes through the activation of SMAD-1/5/8, thus driving bone formation and homeostasis [3,4,5].

The alveolar bone, located in maxilla and mandible, plays a crucial role as the main support for the teeth. Although similar in basic structure to other bone tissues, the alveolar bone undergoes rapid and continuous remodeling due to the eruption of teeth and the functional stresses of chewing [6]. This rapid remodeling process is crucial for the adaptation of tooth position and is influenced by local factors such as growth factors and cytokines as well as systemic factors such as calcitonin and estrogen, which together ensure the maintenance of bone homeostasis [7]. However, they can also contribute to an increased loss of bone volume during bone remodeling after tooth extraction [8,9]. Therefore, various biomaterials are used for bone augmentation to minimize volume loss and achieve bone regeneration following tooth extraction.

Biomaterials for bone regeneration are categorized as autogenous bone, allografts, xenografts and alloplasts [10]. In the last decade, the use of autologous dentin (AD) has also been recognized as a successful biomaterial for bone regeneration in dentistry. It is suitable for bone regeneration as its chemical composition of organic and inorganic substances is most like native bone tissue [11]. The presence of morphogenetic bone proteins in AD indicates its pronounced osteoinductive properties, which primarily distinguishes it from xenogeneic biomaterials that do not contain proteins in their composition [12]. In a recent study in which regenerated bone was histomorphometrically analyzed after the application of autologous dentin, 85% new bone formation and 25% residual dentin were found 7 months after guided bone regeneration (GBR) [13]. Pang et al. [14] histomorphometrically compared the outcome of alveolar ridge preservation using bovine xenograft (BX) and AD, and it was shown that there was no statistically significant difference in bone tissue volume.

Today, cerabone^®^ (botiss biomaterials, Zossen, Germany) is widely used in procedures aimed at regenerating the alveolar ridge. It is derived from trabecular bovine bone, with its organic components, including osteoinducing molecules, immune cells and pathogens, removed by physical and chemical processes, leaving behind calcium hydroxyapatite [15,16].

In the available literature analyzing the osteoconductive properties of xenogeneic biomaterials in a calvarial defect model, only one paper used magnesium in combination with porcine bone [17]. Most published studies have analyzed the percentage of bone volume and remaining biomaterial at two time points [18,19], while very few studies have analyzed bone structural parameters or performed immunohistochemical analyses [18]. Previously, our group of authors published the results of an animal study on the use of four different BX in critical cranial bone defects (CCBDs) of 5 mm in size. The bone samples were analyzed by micro-CT, histology and immunohistochemistry. Overall, the xenogeneic magnesium alloy biomaterial showed key properties of osteoinduction and biodegradability during CCBD healing [20].

However, there is still a knowledge gap regarding the application of magnesium-enriched biomaterials in CCBDs, and, in general, their use in oral surgery is not yet fully understood. Considering the unexplored biological properties and unexplained gene expression of factors involved in bone remodeling during CCBD repair with AD and BX + Mg, the main aim of this study was to investigate them at different time points during the healing of 8 mm CCBDs. Therefore, the relative expression of vascular endothelial growth factor (VEGF), osteocalcin (OC), the receptor activator of nuclear factor κB (RANK) and sclerostin (SOST) were analyzed by real-time polymerase chain reaction (RT-PCR). In addition, the values of the 3D parameters were determined by micro-CT analyses of the bone samples and the expression of osteoinductive proteins and their intercellular messenger molecule were analyzed.

## 2. Materials and Methods

### 2.1. Experimental Animals and Study Design

This study was conducted in accordance with the guidelines of the Declaration of Helsinki and was approved by the Ethics Committee of the University of Rijeka and the Ministry of Agriculture (EP 302/2021).

For this study, 80 male Wistar rats aged around 2.5 months were used. The animals were reared and kept under the laboratory conditions of the Institute of Physiology, Pathophysiology and Immunology of the Faculty of Medicine in Rijeka, fed ad libitum, provided with drinking water and subjected to a daily light and dark cycle in accordance with the regulation on the protection of animals used for scientific purposes (Official Gazette 55/13). The animals were randomly divided into 4 groups of 20 animals each. Each group of animals was named after the name of the material used to promote the healing of the critical calvarial bone defect (CCBD). The animals were sacrificed at 4 different times—on the 7th, 15th, 21st and 30th day of CCBD healing. The data on the test animal groups are listed in Table 1.

### 2.2. Materials

Three different types of biomaterials were used to stimulate the healing of CCBD, namely the following:

1. Cerabone^®^ (botiss GmbH, Zossen, Germany) with magnesium (BX + Mg group), produced at Biotrics Biomiplants AG (Berlin, Germany) in the form of Mg granules in which the mass fraction of the magnesium alloy (a solid solution of magnesium with yttrium, zinc, manganese and calcium) is 3%. Additional information is not available because the biomaterial itself is still the subject of research, and thus all data are confidential.
2. Cerabone^®^ (BX group) made from trabecular bovine bone from which the organic component of the bone tissue was removed by physical and chemical processes.
3. Autologous dentin from rat teeth (AD group) ground with a dentin grinder (KometaBio Smart Dentin Grinder, Tenafly, NJ, USA) and then prepared according to a protocol previously described by our group of authors before being applied to the defect [21]. To collect dentin material for the AD group, tooth extractions were performed on donor inbred Wistar rats six days prior to the CCBD surgery [21,22,23].

After the implantation of the biomaterial, the implant site was covered with a collagen membrane (mucoderm^®^, Botiss Biomaterials, Zossen, Germany). In the control group, only a collagen membrane was used to cover a defect, but no bone biomaterial.

### 2.3. Surgical Protocol and Bone Sample Harvesting

Surgical instruments sterilized in an autoclave and cooled to room temperature were used to perform the CCBD and implant the healing-promoting material. The work surface for performing the surgical procedures on the animal was disinfected once with 70% ethanol.

The animals were anesthetized using ketamine (80 mg/kg) and xylazine (5 mg/kg body weight). For pain relief, an intraperitoneal injection of tramadol (10 mg/kg, Henry Schein, Melville, NY, USA) was administered. A local anesthetic of 0.3–0.4 mL of 1% lidocaine was applied subcutaneously at the incision site. During surgery, each animal received a subcutaneous injection of sterile saline (0.9% NaCl, Henry Schein, NY, USA) at a rate of 10 mL/kg/h to compensate for visible and invisible fluid loss during and after the procedure. Blood oxygen levels were continuously monitored using a pulse oximeter (MouseSTAT, Pulse Oximeter & Heart Rate Monitor Module, Kent Scientific Corporation, Torrington, CT, USA), while the depth of anesthesia and analgesia was assessed by observing the animal’s response to external stimuli. The surgical area on the animal’s head was prepared by shaving the fur with an electric trimmer designed for small animals (MOSER 1556 AKKU, professional cordless hair trimmer, BIOSEB in vivo Research Instruments, Schönwalde-Glien, Germany), covering the region from the muzzle between the eyes to the back of the skull. The area was then sterilized using iodine sticks (Impregnated Swabstick Dynarex 10% Strength Povidone-Iodine Individual Packet, Dynarex, Orangeburg, New York, NY, USA). An incision was made in the skin at the prepared site, followed by the application of a sterile drape. A 1.5 cm periosteal incision was performed across the skull, from the nasal bone to the bregma, exposing the calvarial bone after retracting the periosteum. A trephine with an outer diameter of 8 mm (Helmut Zepf, Seitingen-Oberflacht, Germany) was used to drill the fronto-parietal complex at 1500 rpm, creating an intracranial defect. Throughout the drilling process, sterile saline was applied dropwise, approximately one drop every two seconds, to both the eyelid and calvaria. The low drilling speed and continuous moisturizing were essential to prevent thermal injury that could damage surrounding tissue. Gentle pressure with an elevator was used to lift a part of the bone at the injury edge, detaching it from the underlying dura. The defect site was thoroughly rinsed with sterile physiological solution to clear away bone fragments and dust generated during drilling. To standardize the defect site, a rat head holder (Model 920-E Rat Head Holder, David Kopf Instruments, Los Angeles, CA, USA) and a tissue marking instrument (Biopsy Punch, Kai Medical, Tokyo, Japan) were utilized. Since a trephine with a diameter of 8 mm was used for surgical bone removal and defect formation, the defects themselves were standardized. When drilling into the calvaria, care was taken to ensure that the instrument did not penetrate too deeply. As the thickness of the calvaria of the experimental animals was approximately 1 mm, the markings on the eyelid were used as a guide to depth and the entire drilling process was continuously monitored for depth. To avoid injury to the skull or brain, the force applied was less than the weight of the drill. As the calvaria becomes transparent on the defect during drilling, the dura and the surface of the brain can be more easily recognized. As the defect approached the appropriate thickness, the drilled part of the bone would move slightly downward, indicating that the drilled calvaria had almost reached full thickness. A specific type of biomaterial was then implanted into the surgically created calvarial bone defect (see Table 1). The biomaterial was weighed using a precision balance (ME-T Precision Balance, Mettler Toledo, Columbus, OH, USA) to ensure a consistent amount of 20 mg for each animal. The granulated biomaterial had a uniform particle size of 0.5 to 1 mm across all groups. To complete the procedure, the implantation site was covered with a collagen membrane.

The skin was sutured over the biomaterial and collagen membrane using either single or continuous sutures (3-0 USP, Hu-Friedy Perma Sharp Sutures, Irvine, CA, USA). After completing the procedure, the surgical site was thoroughly cleaned with sterile saline or diluted hydrogen peroxide (3%) to remove any remaining blood. The animals were then placed in cages equipped with heating pads (heating pads for rats—20.5 × 12 cm, DC temperature controller, FHC, South Gate, CA, USA) to warm them up quickly and safely, helping to minimize postoperative trauma. In this way, the animals’ body temperature was permanently maintained in the range of 36.5 to 37.5 °C. The animals in each of the 4 groups were randomly divided into a further 4 groups of 5 animals, depending on the time of sacrifice. The animals were sacrificed at intervals of 7, 15, 21 and 30 days after the operation.

Sacrifice was carried out in the usual humane manner with three times the anesthetic dose, i.e., with ketamine (240 mg/kg) and xylazine (15 mg/kg of body weight). After sacrificing the experimental animals, tissue samples were taken from the entire fronto-parietal–occipital complex at the site of the pre-existing CCBD in which the biomaterial was embedded. Depending on the research method, the tissue was subjected to different preparation methods.

### 2.4. Micro-CT Analysis of 3D Parameters

The tissue samples were preserved in 4% paraformaldehyde at 4 °C prior to transport. Before being transported, the samples were placed in a 70% alcohol solution until they could be scanned using a micro-CT device.

The samples were then scanned with a micro-CT scanner (Skyscan 1076, Bruker, Kontich, Belgium) at a resolution of 18 μm, with a 0.40-degree rotation and a 0.025 mm titanium filter. The average image section was set to 2. The resulting images were reconstructed using NRecon software v. 2.0 (Bruker, Kontich, Belgium) and analyzed with CTAn software v.1.8. (Bruker, Kontich, Belgium). For the analysis, a circular area with a diameter of 8 mm was drawn along the edges of the original defect edge. To distinguish the new bone formation from the used biomaterial, specific thresholds were used for each biomaterial, while they remained the same for the new bone formation. The threshold for BX and BX + Mg was 200–255, and for AD, 110–255, while for new bone formation, it was 50–255. The value range of the applied threshold differentiated the individual biomaterials according to different densities. The value of the biomaterial in relation to new bone formation was then determined by subtraction. Based on that, the following parameters were calculated: the ratio of bone volume to trabecular volume (bone volume/total volume, BV/TV, %) and the percentage of residual biomaterial (RB, %).

### 2.5. Real-Time Polymerase Chain Reaction (RT-PCR) Analysis

The relative expression levels of the following proteins were analyzed using the RT-PCR method on bone samples: vascular endothelial growth factor (VEGF), osteocalcin (OC), receptor activator of nuclear factor κB (RANK) and sclerostin (SOST). The protocols recommended by the manufacturer were followed when performing the RT-PCR method.

RNA isolation was performed as follows. Tissue samples from rat calvarial halves with defect and biomaterial were stored at −80 °C after sacrifice. On the day of isolation, the tissue samples were taken at −80 °C and placed in liquid nitrogen. During the mechanical homogenization of the bone in the crucible, the bone was cooled with liquid nitrogen, after which 30 mg of bone tissue from each sample was separated into each individual 1.5 mL test tube and then refrozen in liquid nitrogen. The “mini” NucleoSpin^®^ RNA protocol (Macherey-Nagel GmbH & Co. KG, Düren, Germany) was used for RNA isolation. Complementary DNA (cDNA) was obtained by the reverse transcription of total RNA using the High Capacity cDNA Reverse Transcription Kit (Applied Biosystems, Foster City, CA, USA). The amount of messenger RNA for VEGF, RANK, SOST and OC primers was determined with specific Taqman probes using the 7300 Fast Real-Time PCR System (Applied Biosystems, Foster City, CA, USA).

A reaction in which water was added instead of reverse transcriptase (“no RT control”, NRTC) was used as a negative control that excluded the multiplication of genomic DNA. Contamination with exogenous nucleic acids was excluded using a control in which the cDNA was replaced with water. Amplification was performed with the 7300 Real Time PCR System under standardized conditions: incubation, 50 °C/2 min/1 cycle; initial denaturation, 95 °C/10 min/1 cycle; and 40 amplification cycles at 95 °C for 15 s (denaturation) and at 60 °C for 1 min (maturation/elongation). The amplification results were analyzed using the 7300 System SDS Software v1.3 computer application. The reactions of all samples were performed in duplicate, and the result was considered reliable only when there were no differences between the amplification curves.

The comparative cycle threshold (CT) method (∆∆CT) was used for the relative representation of gene expression according to the following formula: fold-change (FC) = 2^−∆∆CT^ [24]. The results are presented as a relative change in gene expression compared to the control sample. The abbreviation CT stands for the cycle in which the signal of the target genes rises above the detection limit for the first time. The first delta in the formula indicates the difference between the values for the housekeeping gene and the genes of interest, i.e., the level of gene transcriptional activity of each sample normalized with the values obtained for the housekeeping gene GAPDH used as endogenous control for the sample of interest, while the second delta indicates the difference between the values of the treated group and the untreated control groups.

### 2.6. Immunohistochemical Analysis

The expression of protetctive cytokines (VEGF) and osteoinductive proteins and their intracellular messenger molecules (BMP-2/4 and SMAD-1/5/8) was determined by immunohistochemical analysis.

The primary antibodies listed below were used for the immunohistochemical analyzes:

- BMP-2/4 (sc-137087 SCBT, Santa Cruz Biotechnology, Dallas, TX, USA, SAD), 1:200;
- VEGF (ab 231260 abcam, Abcam, Cambridge, UK), 1:200;
- SMAD-1/5/8 (#95115 CST, Cell Signaling Technology, Danvers, MA, USA, SAD), 1:100.

The secondary antibodies were obtained from the manufacturer Dako (2° Real En Vision Detection System rabbit/mouse, Glostrup, Denmark). The tissue sections were first deparaffinized using a xylene solution and then dehydrated through a series of ethyl alcohol solutions with decreasing concentrations (100%, 96% and 75%). Next, the sections were washed three times with PBS solution and heated in a 10 mM citrate buffer (pH 6.0) at 65 °C for 20 min. Afterward, the sections were washed with a 0.3% Triton X-100 solution in PBS at room temperature. Endogenous peroxidase was then inactivated by treating the sections with 0.3% hydrogen peroxide in methanol for 30 min. Following a PBS wash, the tissue sections were treated with 10% normal serum for 60 min, chosen according to the species of the secondary antibody carrier. The primary antibodies dissolved in PBS solution were incubated in a humid chamber at a temperature of 4 °C for 18 h. After washing the tissue sections in PBS solution (pH 7.4), a secondary antibody was added depending on the primary antibody. After 60 min, the tissue section was washed in PBS solution and treated with streptavidin. 3,3-diaminobenzidine in hydrogen peroxide (DAB) was used for visualization. The tissue section was then washed with water and counterstained with hematoxylin. The tissue was rehydrated in ethyl alcohols of increasing concentrations (75%, 96% and 100%). After clarification with xylene, the specimen was placed in entalan. The intensity of the immunohistochemical staining was evaluated with the computer program ImageJ v 1.54 (available from: https://imagej.net/ij/).

### 2.7. Statistical Analysis

Statistical analysis was carried out using the Statistica 11.1 software (StatSoft, Inc., Tulsa, OK, USA). After confirming the normal distribution of the data, differences between the biomaterial groups were assessed using repeated measures analysis of variance (ANOVA). Tukey’s Highly Significant Difference Test was employed for post hoc analysis to identify specific differences between the groups. Additionally, multiple regression analysis was conducted to evaluate the impact of predictor variables on bone volume. The results were considered statistically significant at *p* < 0.01 and *p* < 0.05.

## 3. Results

### 3.1. Mirco-CT Analysis

**Figure 1:**
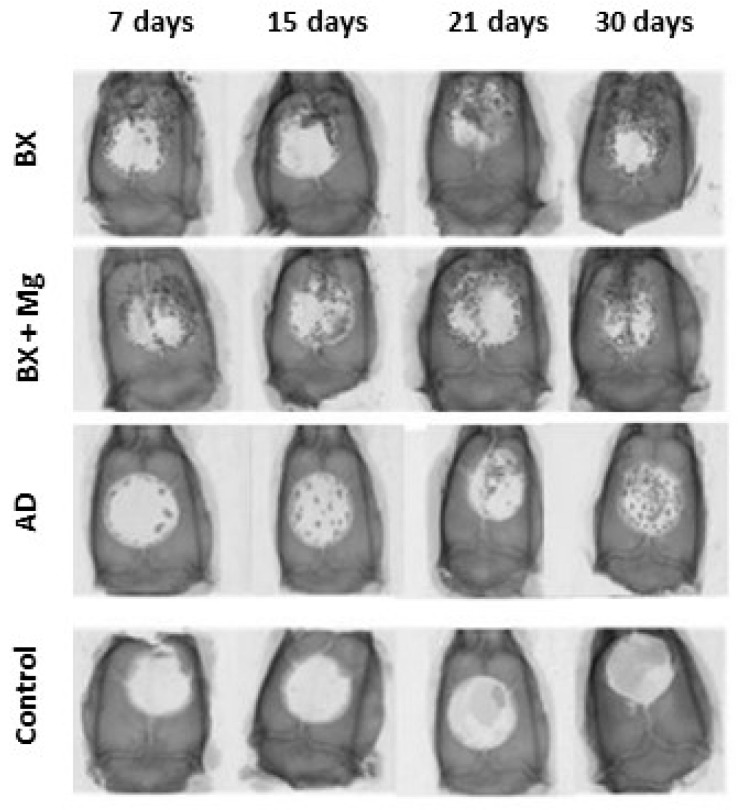
Visualization of the fronto-parieto-occipital complex of the calvaria of rats, imaged with a micro-CT device. The images show the progression of CCBD closure over the days of healing.

A series of images of the calvaria of animals from different groups and on different days of healing clearly shows the extent of CCBD closure (Figure 1).

The quantitative results of the micro-CT analysis are summarized in Table 2. They show statistically significant differences in the micro-CT bone morphometry parameters depending on the biomaterial and the day. In general, the values of bone volume measured by micro-CT increased with increasing time intervals, while the values of residual biomaterial decreased during the healing period. On the 30th day of healing, the AD group had the highest bone volume and the lowest residual biomaterial values.

### 3.2. The Results of Real-Time Polymerase Chain Reaction (RT-PCR) Analysis

**Figure 2:**
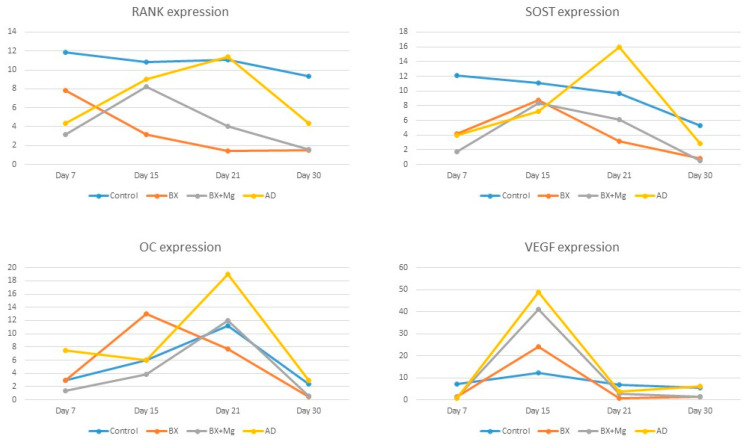
The expressions of RANK, SOST and OC were highest in the AD group on day 21 of healing. The VEGF expression was also highest in the AD group, but on the 15th day of healing.

Figure 2 summarizes the results of the gene expression of the bone remodeling factors osteocalcin (OC), receptor activator of nuclear factor κB (RANK) and sclerostin (SOST), as well as the gene expression of the vascular endothelial growth factor (VEGF).

### 3.3. Results of Immunohistochemical Analysis

#### 3.3.1. Immunohistochemical Analysis of BMP-2/4 Expression

**Figure 3:**
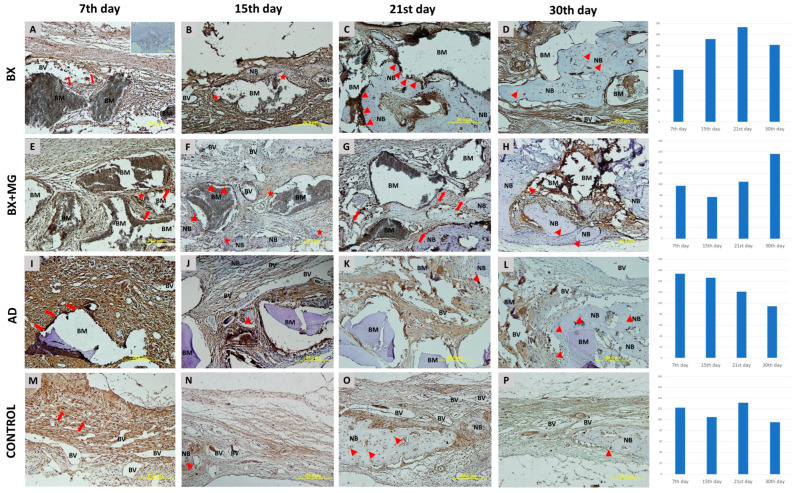
BMP-2/4 immunohistochemical staining of coronal sections of calvarial bone defects in the following groups: BX—bovine xenograft (**A**–**D**); BX + MG—bovine xenograft and magnesium (**E**–**H**); AD—autologous dentin (**I**–**L**); and control group (**M**–**P**) on the 7th, 15th, 21st and 30th day. Legend of abbreviations. The BMP-2/4 immunohistochemical staining of the coronal sections of the calvarial bone defects are shown for the following groups: BX—bovine xenograft (**A**–**D**); BX + MG—bovine xenograft; BM—biomaterial; NB—new bone formation; BV—blood vessel. Triangles (▲) indicate osteoblasts and osteocytes in lacunae, asterisks (★) indicate immunopositive multinucleated cells, and arrows (↑) indicate sites of bone bridging and apposition. On the right side of the immunohistochemistry slides, there are diagrams showing the immunohistochemical staining for each biomaterial broken down by day, on days 7, 15, 21 and 30 of healing.

More intense BMP-2/4 immunopositivity was observed in mesenchymal cells in transition during the earlier stages of healing. From mid-healing to later stages, there are numerous immunopositive osteoblasts, their precursors and osteocytes. Multinucleated cells are also visible, appearing on day 7 in the BX group and on days 7, 15 and 21 in the BX + Mg group (Figure 3). The relative values of immunohistochemical BMP-2/4 staining intensity for each biomaterial examined are shown in Figure 3. The general trend shows the highest values in the BX group, with the highest single value measured on day 21 of healing (r = 173.419).

#### 3.3.2. Immunohistochemical Analysis of SMAD-1/5/8 Expression

**Figure 4:**
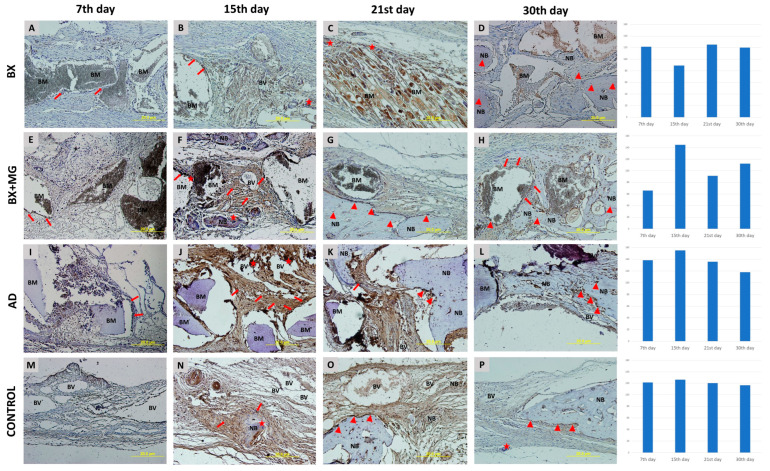
SMAD-1/5/8 immunohistochemical staining of coronal sections of calvarial bone defects for the following groups: BX—bovine xenograft (**A**–**D**); BX + MG—bovine xenograft and magnesium (**E**–**H**); AD—autologous dentin (**I**–**L**); and control group (**M**–**P**) on the 7th, 15th, 21st and 30th day. Legend of abbreviations: BM—biomaterial; NB—new bone formation; BV—blood vessel. Triangles (▲) indicate osteoblasts and osteocytes in lacunae, asterisks (★) indicate immunopositive multinucleated cells, and arrows (↑) indicate sites of bone bridging and apposition. On the right side of the immunohistochemistry slides, there are diagrams showing the immunohistochemical staining for each biomaterial broken down by day, on days 7, 15, 21 and 30 of healing.

SMAD-1/5/8 intracellular signaling molecules track the expression of BMP-2/4. Similar to the immunolocalization observed for BMP-2/4, SMAD-1/5/8 is present in mesenchymal cells, osteoblasts and osteocytes and is also found in multinucleated cells, sporadically in connective tissue and around degrading biomaterial particles (Figure 4). The relative values of the immunohistochemical staining intensity of SMAD-1/5/8 for each biomaterial examined are shown in Figure 4. The general trend indicates the highest immunohistochemical staining values in the AD group, with the highest single value recorded on the 15th day of healing (r = 155.404).

#### 3.3.3. Immunohistochemical Analysis of VEGF Expression

**Figure 5:**
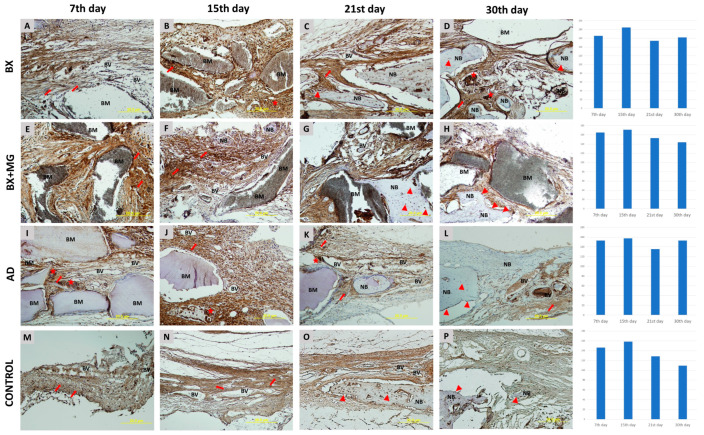
VEGF immunohistochemical staining of coronal sections of calvarial bone defects for the following groups: BX—bovine xenograft (**A**–**D**); BX + MG—bovine xenograft and magnesium (**E**–**H**); AD—autologous dentin (**I**–**L**); and control group (**M**–**P**) on the 7th, 15th, 21st and 30th day. Legend of abbreviations: BM—biomaterial; NB—new bone formation; BV—blood vessel. Triangles (▲) indicate osteoblasts and osteocytes in lacunae, asterisks (★) indicate immunopositive multinucle-ated cells, and arrows (↑) indicate sites of bone bridging and apposition. On the right side of the immunohistochemistry slides; there are diagrams showing the immunohistochemical staining for each biomaterial broken down by day, on days 7, 15, 21 and 30 of healing.

A visible expression of the VEGF is observed in pluripotent mesenchymal cells at sites of neovascularization and at sites where blood vessels infiltrate biomaterial particles that are degraded (Figure 5). The relative values of the immunohistochemical VEGF staining intensity for each biomaterial examined are shown in Figure 5. The general trend indicates the highest immunohistochemical staining values in the BX group, with the highest single value recorded on day 15 of healing (r = 184.955).

## 4. Discussion

This study investigated the osteoconductive and osteoinductive properties of different biomaterials in models of critical cranial bone defects (CCBDs). The focus was on the comparison of bovine xenograft (BX), bovine xenograft with magnesium (BX + Mg) and autologous dentin (AD) for bone regeneration using micro-CT and immunohistochemical analysis, as well as on the expression of key genes involved in bone remodeling.

The micro-CT analysis generally demonstrated that all biomaterials promoted bone formation over time, with AD showing the highest bone volume (BV/TV) and the lowest residual biomaterial (RB) on the 30th day of healing (Table 2). The AD group showed statistically significantly higher BV/TV values on day 21 than the BX, BX + Mg and control groups. When analyzing the healing dynamics, we observed the highest bone growth in the AD group between day 15 and 21, which means that the bone is intensively formed and remodeled during this period. The basis for this statement lies in the fact that AD has both osteoconductive and osteoinductive properties [25]. In this context, AD as an implantation material in bone defects is characterized by the presence of morphogenetic bone proteins that promote bone formation. Therefore, it has very similar properties to native bone in terms of both organic and inorganic substances [26,27].

The addition of magnesium to bovine xenograft (BX + Mg) resulted in significantly higher bone volume compared to BX alone, especially at day 30, suggesting that magnesium enhances the regenerative properties of BX by creating a favorable environment for bone tissue formation and possibly influencing the activity of osteoblasts and osteoclasts, thus promoting balanced bone remodeling (Table 2). The use of magnesium implants found its basis in clinical research as early as the 1930s [28]. The complete resorption of magnesium and the short postoperative recovery without pain encouraged Lambotte to pioneer the use of magnesium implants for supracondylar fractures in children, as they healed quickly. Although magnesium implants are degraded in vivo by corrosion, they have no harmful effects on the tissue. On the contrary, they stimulate osteoblast activity around the implant, leading to the complete replacement of the implant by bone tissue [29,30].

The study by Perić Kačarević et al. [31] investigated magnesium implants and their biological interactions with bone tissue. Their research revealed that the calcium phosphate corrosive layer that forms on magnesium implants after implantation helps to slow down the corrosion process, allowing direct contact with bone tissue. During corrosion, metallic magnesium undergoes oxidation, releasing magnesium ions as corrosion products. This process causes the protective layer of magnesium hydroxide to dissolve, at least in localized areas, which permits the corrosion to continue until the implant is fully degraded. The study also indicated that an alkaline pH is unlikely to negatively impact bone regeneration, as bone graft materials made of pure hydroxyapatite decompose at slightly alkaline pH levels and have been effectively used in bone regeneration for many years. Similar findings were found by Jung et al. [32]. The biomaterial was almost completely resorbed within only 3 months. In a recent experimental study, a magnesium alloy-enriched biomaterial was implanted in the distal condyle of an animal. After three months, the magnesium-enriched material had decomposed, with much of the original magnesium alloy having disappeared. Simultaneously, a fibrous capsule had formed around the surgical site. Histological analysis showed that the magnesium scaffolds had not caused any significant damage to the surrounding tissue. This study shows that even fast-degrading magnesium scaffolds maintain good biocompatibility and trigger an appropriate inflammatory response in vivo. Consequently, magnesium alloy-based implants hold promise for applications in oral implantology [33].

Based on the available literature investigating the osteoconductive properties of magnesium-enriched xenogeneic biomaterials, our study on a 5 mm calvarial defect stands out with the highest bone volume values for the BX + Mg group in all observed time intervals compared to the other two BX groups [20]. This finding can be fully equated with the results for a 8 mm defect. The observed residual biomaterial is lower in the BX + Mg group than in the BX group, suggesting also that magnesium contributes to a better degradation of the biomaterial.

There is also a publication in which magnesium was used in combination with porcine bone [17]. In the calvaria of 14 adult male New Zealand rabbits, defects with a diameter of 7 mm were created and filled with the following biomaterials: untreated porcine bone, BioOss^®^ (Geistlich, Wolhusen, Switzerland) and porcine bone containing Mg. The percentage of new bone formation was analyzed histomorphometrically 2 and 4 weeks after implantation. The results showed that in the magnesium-filled group, the percentage of new bone formation was 11.8% at 2 weeks and 22.3% at 4 weeks, with statistically significantly higher results in this group compared to the other two groups. However, we cannot objectively compare our study with the aforementioned study because we did not analyze our samples histomorphometrically.

The RT-PCR results offer insights into the molecular mechanisms involved in bone regeneration. The analysis focused on the relative expression levels of vascular endothelial growth factor (VEGF), osteocalcin (OC), receptor activator of nuclear factor κB (RANK) and sclerostin (SOST). VEGF, which plays a key role in angiogenesis, was found to be upregulated in the AD and BX + Mg groups, indicating enhanced vascularization—a crucial factor for bone healing and regeneration [34]. In the study conducted by Hassumi and colleagues, the relative level of gene expression for several proteins in the alveolar bone was analyzed by RT-PCR [35]. The levels of these genes were compared on days 7, 14 and 28. RANK was found to have the highest value on day 28, which is comparable to our finding that RANK in the AD group reaches the highest value on day 21 (Figure 2), while in the other groups, the highest values are recorded in the middle of the healing process (day 15). In addition, in the abovementioned study, the highest levels of gene expression for OC were found on day 28. Our study shows that the highest gene expression levels of OC are found in the AD group on day 21 (Figure 2). The BX + Mg and the control groups also showed the highest gene expression levels of OC on day 21, and the BX group, on day 15. Osteocalcin is a protein secreted by mature osteoblasts, and since it binds to calcium, it is the best indicator of bone formation [36]. This result indicates that most bone tissue is formed between days 15 and 21. The lower expression of SOST, an inhibitor of bone formation, in the AD group is consistent with the higher bone volume observed (Table 2) in this group, as this indicates less inhibition of osteogenesis.

Immunohistochemical analysis for VEGF, BMP-2/4 and SMAD-1/5/8 proteins provided further confirmation of the molecular findings. Following the increased stimulation of osteoblasts, SMAD-1/5/8 are activated in the BMP signaling pathway and promote osteogenesis by regulating osteogenic genes [5]. The expression of SMAD-1/5/8 follows the expression of BMP-7, which was confirmed by our results where the tested biomaterials showed an increase in levels in the middle of the healing cycle, with the highest levels occurring in the AD group on day 15. VEGF promotes neovascularization and is expressed at low levels at the beginning of osteoblast differentiation, then strongly during terminal differentiation, and reaches a maximum during the mineralization process [37]. In all biomaterials tested, an increase in the measured values was observed in the middle of the healing cycle, with the highest measured value in the BX group on day 15. In the study conducted by Pires et al. [38], the effectiveness of synthetic hydroxyapatite and xenografts, both in their pure form and enriched with the mononuclear fraction of bone marrow, was compared for the regeneration of critical-sized bone defects in the calvaria of rats. This comparison was made using histomorphometric and immunohistochemical analyses (anti-VEGF, anti-osteopontin). Forty rats were divided into five groups based on the biomaterials used: synthetic hydroxyapatite, xenograft, synthetic hydroxyapatite enriched with the mononuclear fraction of bone marrow, xenograft enriched with the mononuclear fraction and a control group with no intervention. The animals had 8 mm critical-sized bone defects created, and after eight weeks, they were euthanized. The data analysis revealed a significant increase in new bone matrix formation in all experimental groups compared to the control group [38].

In this study, we chose micro-CT analysis to evaluate the quantitative bone parameters because of its high sensitivity and accuracy in quantifying bone volume and residual biomaterial [39]. The parameters evaluated, including BV/TV and RB, are widely recognized indicators of bone regeneration and provide a reliable measure of the efficacy of the materials tested. While histochemical analysis could have provided complementary information, the quantitative nature of micro-CT provides a comprehensive assessment that meets the aims of this research. Future studies could incorporate histologic analysis alongside the quantitative data from micro-CT to further improve the understanding of bone tissue properties.

It is important to consider the possible impact of tooth extraction on the immune response in the AD group. It is known that any wound that occurs, such as after a tooth extraction, triggers an acute immune response that could activate the wound healing pathways and promote tissue regeneration [40]. This immune activation, which occurred immediately prior to CCBD formation, may have influenced the regenerative environment and contributed to the enhanced healing observed in this group. In particular, the release of cytokines and the recruitment of immune cells after extraction may have accelerated the early stages of bone repair [40,41]. Although this potential influence is consistent with our findings, further studies would be required to specifically investigate the role of such immune modulation in combination with AD grafts. Another focus of future research could be the evaluation of the gene expression of genes of the early healing phase, such as runt-related transcription factor 2 (RUNX2), Osterix, etc. These genes play a crucial role in the early stages of bone formation and could provide a more precise understanding of the timing of bone healing [42]. Expanding gene analysis in future studies could lead to a more comprehensive characterization of the molecular events underlying the healing process.

In conclusion, this study provides comprehensive evidence of the osteogenic properties of AD and BX + Mg in a CCBD model. The increased bone volume, reduced amount of residual biomaterial and favorable gene expression profiles highlight the potential of BX + Mg for clinical application.

## Figures and Tables

**Table 1:** Presentation of the distribution of experimental animals according to the group and experimental duration of CCBD healing.

Group Name	Number of Animals	Time Points	Total
BX ^1^	5	7th, 15th, 21st and 30th day	20
BX + Mg ^2^	5	7th, 15th, 21st and 30th day	20
AD ^3^	5	7th, 15th, 21st and 30th day	20
Control	5	7th, 15th, 21st and 30th day	20
Total			80

^1^ Bovine xenograft, ^2^ bovine xenograft and magnesium, ^3^ autologous dentin.

**Table 2:** The quantitative results of the micro-CT analysis.

		Biomaterials			
		BX ^3^ (N = 5)	BX + Mg ^4^ (N = 5)	AD ^5^ (N = 5)	Control ^6^ (N = 5)
Days	μCT Parametrs	Mean ± SD	Mean ± SD	Mean ± SD	Mean ± SD
7	BV/TV ^1^ (%)	19,795 ± 1943 ^a,g^	19,813 ± 1643 ^a,e,g^	18,935 ± 1601 ^a,g^	6952 ± 1002 ^f^
	RB ^2^ (%)	27,847 ± 1468 ^f^	24,626 ± 1520 ^f^	29,459 ± 1164 ^c,g^	/
15	BV/TV (%)	24,238 ± 1943 ^a,f^	23,848 ± 1643 ^a,f^	24,967 ± 1601 ^a,g^	9906 ± 1002
	RB (%)	30,989 ± 1468 ^b,c,g^	20,904 ± 1520 ^f^	22,886 ± 1164 ^g^	/
21	BV/TV (%)	31,033 ± 1943 ^a^	31,885 ± 1643 ^a^	37,909 ± 1601 ^a,c,f^	11,921 ± 1002
	RB (%)	22,912 ± 1468 ^b^	21,986 ± 1520 ^f,b^	12,916 ± 1164	/
30	BV/TV (%)	33,015 ± 1943 ^a^	39,501 ± 1643 ^a,d^	48,994 ± 1601 ^a,c^	16,938 ± 1002
	RB (%)	19,905 ± 1468 ^b,c^	12,726 ± 1520	11,996 ± 1164	/

Legend: ^1^ bone volume/total volume; ^2^ residual biomaterial; ^3^ bovine xenograft; ^4^ bovine xenograft + magnesium; ^5^ autologous dentin; ^6^ control. Statistical significance (*p* < 0.05) compared to ^a^ control; ^b^ AD; ^c^ BX + Mg; ^d^ BX; ^e^ 15th day; ^f^ 30th day; ^g^ 21st and 30th day.

## Data Availability

The data presented in this article are available upon request from the corresponding author.
